# The Ubiquitin E3 Ligase Parkin Inhibits Innate Antiviral Immunity Through K48-Linked Polyubiquitination of RIG-I and MDA5

**DOI:** 10.3389/fimmu.2020.01926

**Published:** 2020-09-02

**Authors:** Lang Bu, Huan Wang, Panpan Hou, Shuting Guo, Miao He, Jingshu Xiao, Ping Li, Yongheng Zhong, Penghui Jia, Yuanyuan Cao, Guanzhan Liang, Chenwei Yang, Lang Chen, Deyin Guo, Chun-Mei Li

**Affiliations:** ^1^MOE Key Laboratory of Tropical Disease Control, the Infection and Immunity Center (TIIC), School of Medicine, Sun Yat-sen University, Shenzhen, China; ^2^Institute of Precision Medicine, The First Affiliated Hospital, Sun Yat-sen University, Guangzhou, China; ^3^Department of Immunology, School of Basic Medical Sciences, Wuhan University, Wuhan, China; ^4^School of Basic Medical Sciences, Anhui Medical University, Hefei, China

**Keywords:** parkin, mitophagy, polyubiquitination, RIG-I, MDA5, innate immunity

## Abstract

Innate immunity is the first-line defense against antiviral or antimicrobial infection. RIG-I and MDA5, which mediate the recognition of pathogen-derived nucleic acids, are essential for production of type I interferons (IFN). Here, we identified mitochondrion depolarization inducer carbonyl cyanide 3-chlorophenylhydrazone (CCCP) inhibited the response and antiviral activity of type I IFN during viral infection. Furthermore, we found that the PTEN-induced putative kinase 1 (PINK1) and the E3 ubiquitin-protein ligase Parkin mediated mitophagy, thus negatively regulating the activation of RIG-I and MDA5. Parkin directly interacted with and catalyzed the K48-linked polyubiquitination and subsequent degradation of RIG-I and MDA5. Thus, we demonstrate that Parkin limits RLR-triggered innate immunity activation, suggesting Parkin as a potential therapeutic target for the control of viral infection.

## Introduction

The self- vs. non-self-recognition by the innate immune system has been first reported by Charles Alderson Janeway in 1989 (1). Janeway proposed that the so-called pathogen-associated molecular patterns (PAMPs), which are conserved structures in microorganisms, were recognized as non-self by a germline-encoded pattern-recognition receptor (PRR) system (1). Recent evidences indicate that PRRs are also responsible for recognizing the damage-associated molecular patterns (DAMPs) released from damaged cells. Till now, different classes of PRRs have been reported, including Toll-like receptors (TLRs), nucleotide oligomerization domain (NOD)-like receptors (NLRs), C-type lectin receptors (CLRs), and retinoic acid-inducible gene I (RIG-I)-like receptors (RLRs) (2–6).

The RLR family comprises three members: RIG-I, MDA5, and laboratory of genetics and physiology 2 (LGP2). MDA5 and RIG-I both bind to dsRNA (7). They all have a DExD/H box RNA helicase domain that can detect viral RNA and a C-terminal RNA binding domain (CTD). Both RIG-I and MDA5 contain two caspase activation and recruitment domains (CARDs) in N-terminal region, whereas LGP2 lacks the CARD domains. When the RIG-I or MDA5 recognizes the viral dsRNA, it exposes the N-terminal CARD domains, which interact with the CARD domain of the mitochondrial antiviral signaling protein (MAVS) (8). And then MAVS activates the IKKε and TBK1, which promote the activation of IFN-regulatory factors (IRFs) and nuclear factor-κB (NF-κB) (9). NF-κB and IRFs translocate into the nucleus, where they activate the innate immunity and promote the expression of pro-inflammatory cytokines and type I IFNs (10).

Mitophagy is a special type of mitochondrial autophagy, which can remove damaged or depolarized mitochondria (11, 12). Cells maintain energy balance and resist oxidative stress by regulating the movement, distribution, and clearance of mitochondria (13). Parkinson's disease (PD) is one of the most common neurodegenerative diseases. Although the pathogenesis of PD is still unclear, more and more evidences show that mitochondrial dysfunction is one of the molecular pathogenesis of PD (11, 14). Familial PD can be induced by the mutations of the PTEN-induced putative kinase 1 (PINK1) and E3 ubiquitin-protein ligase Parkin, both of which maintain mitochondrial health by regulating mitochondrial dynamics and quality control (15–17). In normal mitochondria, PINK1 contains a mitochondrial targeting sequence and is transferred from the outer mitochondrial membrane (OMM) to the inner mitochondrial membrane (IMM), where it is cleaved by the protease presenilin-associated rhomboid-like protein (PARL) and degraded by the proteasome 26S subsequently (18, 19). When mitochondria get damaged and depolarized, PINK1 cannot be processed by PARL and stabilized on the OMM. Then, PINK1 can phosphorylate itself and enhance its own activation (20). Furthermore, PINK1 phosphorylates ubiquitin on Ser65, and then Ub-pSer65 binds to Parkin (21). Then Parkin can be phosphorylated by PINK1 on its Ser65, leading to its full activation (22). Once activated, Parkin conjugates ubiquitin chains on OMM proteins and recruits several substrates in mitochondria, such as mitofusin 2 (Mfn2), voltage-dependent anion-selective channel protein (VDAC), and dynamin-1-like protein (DRP1) (23–26). Mitophagy receptors containing a LIR (LC3 interacting region) motif can be recruited by OMM proteins to interact with the LC3 anchored in the membrane of autophagosome. These ubiquitin chains bind to autophagic cargo receptors such as optineurin (OPTN) and sequestosome 1 (SQSTM1, also known as p62), which act in concert with the general autophagy mechanism to capture damaged mitochondria in the autophagosomal double-membrane (27, 28). The fusion with lysosomes facilitates the degradation of mitochondria by lysosomal hydrolases (29). The degradation of mitochondria by this process is referred to as PINK1/Parkin-dependent mitophagy. Mitochondria plays the pivotal role in the innate immune signaling pathway, and mitophagy is a key regulatory mechanism to limit excessive inflammation and maintain tissue homeostasis (30).

In this study, we demonstrated that mitochondria depolarization inducer carbonyl cyanide 3-chlorophenylhydrazone (CCCP) inhibited the antiviral responses and the activity of type I IFN during viral infection. Moreover, we identified that a mitophagy associated protein, the E3 ubiquitin ligase Parkin, was a negative regulator of innate immunity. PINK1 and Parkin mediated mitophagy, which negatively regulated the response of type I IFN. Overall, Parkin interacted with and promoted the K48-linked polyubiquitination of RIG-I and MDA5. Thus Parkin-mediated K48-linked polyubiquitination facilitates the degradation of RIG-I and MDA5.

## Results

### Mitochondrial Uncoupler CCCP Inhibits Type I IFN Responses

**Figure 1:**
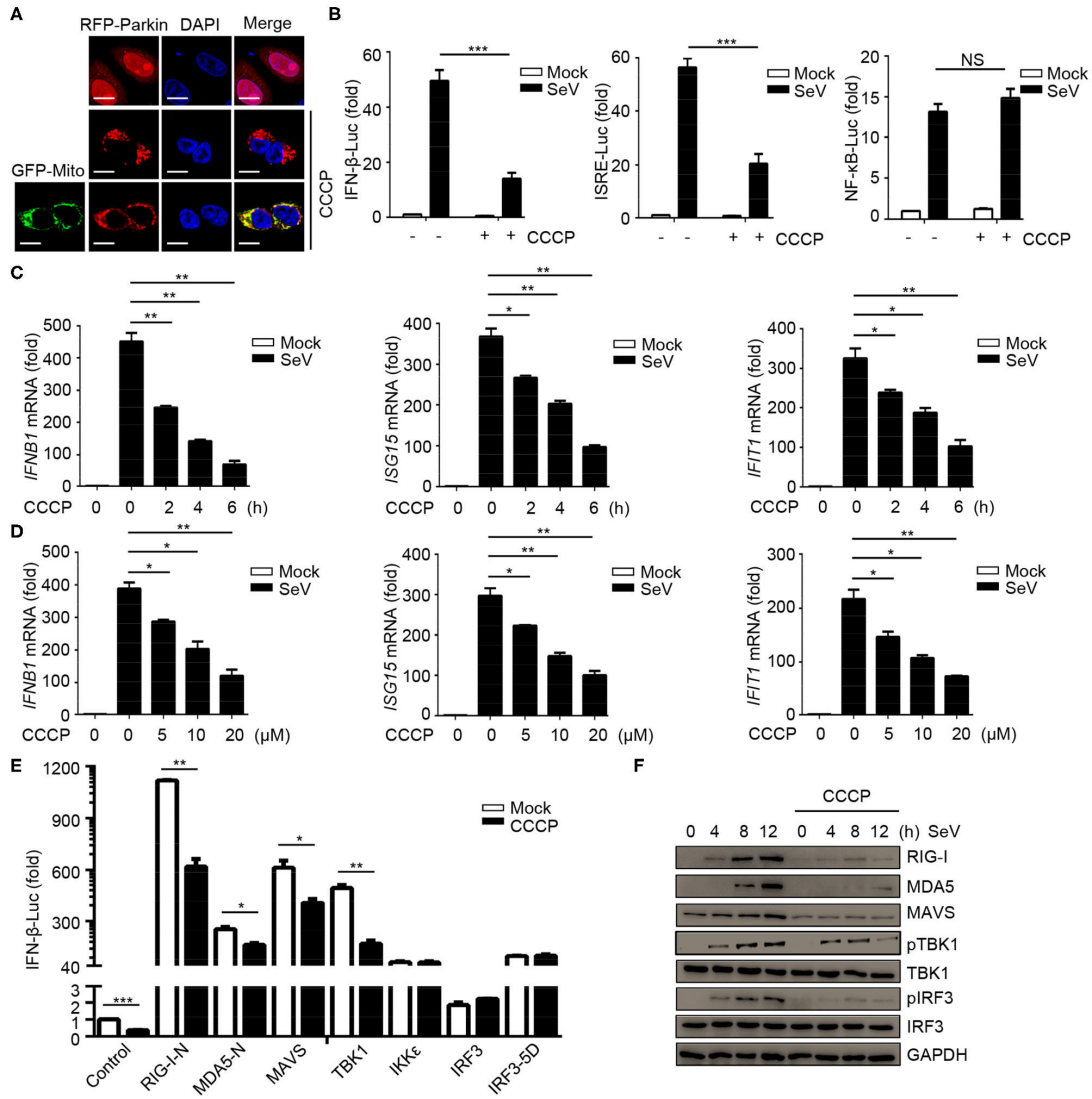
Mitochondrial uncoupler CCCP inhibits type I IFN responses. **(A)** 293T cells were transfected with RFP-Parkin alone or together with GFP-Mito, 24 h post-transfection, the cells were untreated or treated with CCCP (10 μM) for 6 h and subjected to IF analysis. Scale bar is 10 μm. **(B)** HEK293 cells were transfected with a luciferase reporter of the IFN-β promoter, ISRE promoter, and NF-κB promoter. 24 h post-transfection, the cells were untreated or treated with CCCP (10 μM) for 2 h, then left unstimulated or stimulated with SeV for 10 h, and luciferase assays were performed. **(C)** HEK293 cells were untreated or treated with CCCP (10 μM) for different times, unstimulated or stimulated with SeV for 10 h. Then quantitative RT-PCR analysis of *IFNB1, ISG15*, and *IFIT1* mRNA. **(D)** HEK293 cells were untreated or treated with different concentrations of CCCP for 2 h, then unstimulated or stimulated with SeV for 10 h, and subjected to quantitative RT-PCR analysis. **(E)** HEK293 cells were co-transfected with IFN-β luciferase reporter and RIG-I-N, MDA5-N, MAVS, TBK1, IKKε, IRF3, or IRF3-5D for 24 h, then untreated or treated with CCCP (10 μM) for 12 h, and subjected to luciferase assays. **(F)** HEK293 cells were untreated or treated with CCCP (10 μM) for 2 h, then unstimulated or stimulated with SeV for different times, and the whole cell extracts were subjected to IB analysis. The data represent the average of three independent experiments and were analyzed by unpaired *t-*test. All data represent the mean ± S.D. *p < 0.05, **p < 0.01, and ***p < 0.001.

In order to study the possible regulatory effects of mitophagy on innate immunity, we first tested the effect of the carbonyl cyanide 3-chlorophenylhydrazone (CCCP) on mitochondria depolarization. The results showed that Parkin expressed diffusely in the cytoplasm in CCCP untreated cells, while in CCCP treated cells, it was recruited to puncta that co-localized with mitochondria, as monitored by GFP-mito (a mitochondrion-targeted green fluorescent protein) (Figure 1A). In order to reveal, the roles of mitophagy in innate immunity and antiviral responses, we first observed whether mitochondrial uncoupler CCCP inhibited type I IFN responses during viral infection. Luciferase assays showed that CCCP inhibited the SeV-induced activation of *IFNB1* promoter reporter and interferon-stimulating regulatory element (ISRE) promoter reporter containing IRF3-responsive positive regulatory domains (PRD) III and I, but did not inhibit the activation of NF-κB promoter reporter (Figure 1B). These results showed that CCCP inhibited the activation of IFN-β luciferase through IRF3 instead of NF-κB. To confirm the function of CCCP in IFN-β signaling, we detected the expression of *IFNB1* and the transcription of IRF3-dependent genes such as *ISG15* and *IFIT1* mRNA. We observed CCCP decreased the expression of these genes in a dose- and time-dependent manner in HEK293 and Raw264.7 cells (Figures 1C,D and Supplementary Figures 1A,B). Furthermore, CCCP could decrease the expression of the interferon-regulating genes such as *Ifnb1, Isg15*, and *Ifit1* in BMDCs (Supplementary Figure 1C). Then we investigated which step of activation of IFN-β can be influenced by CCCP, the results of which indicated that CCCP inhibited the *IFNB1* promoter reporter induced by upstream activators, including RIG-I-N, MDA5-N, MAVS, and TBK1 (Figure 1E). Similarly, CCCP decreased the expression of SeV-induced RIG-I, MDA5, MAVS, and the phosphorylation of TBK1 and IRF3 (Figure 1F). All together, these data demonstrate that CCCP can inhibit SeV-induced interferon responses.

### E3 Ubiquitin Ligase Parkin Negatively Regulates the Responses and Antiviral Immunity of Type I IFN

**Figure 2:**
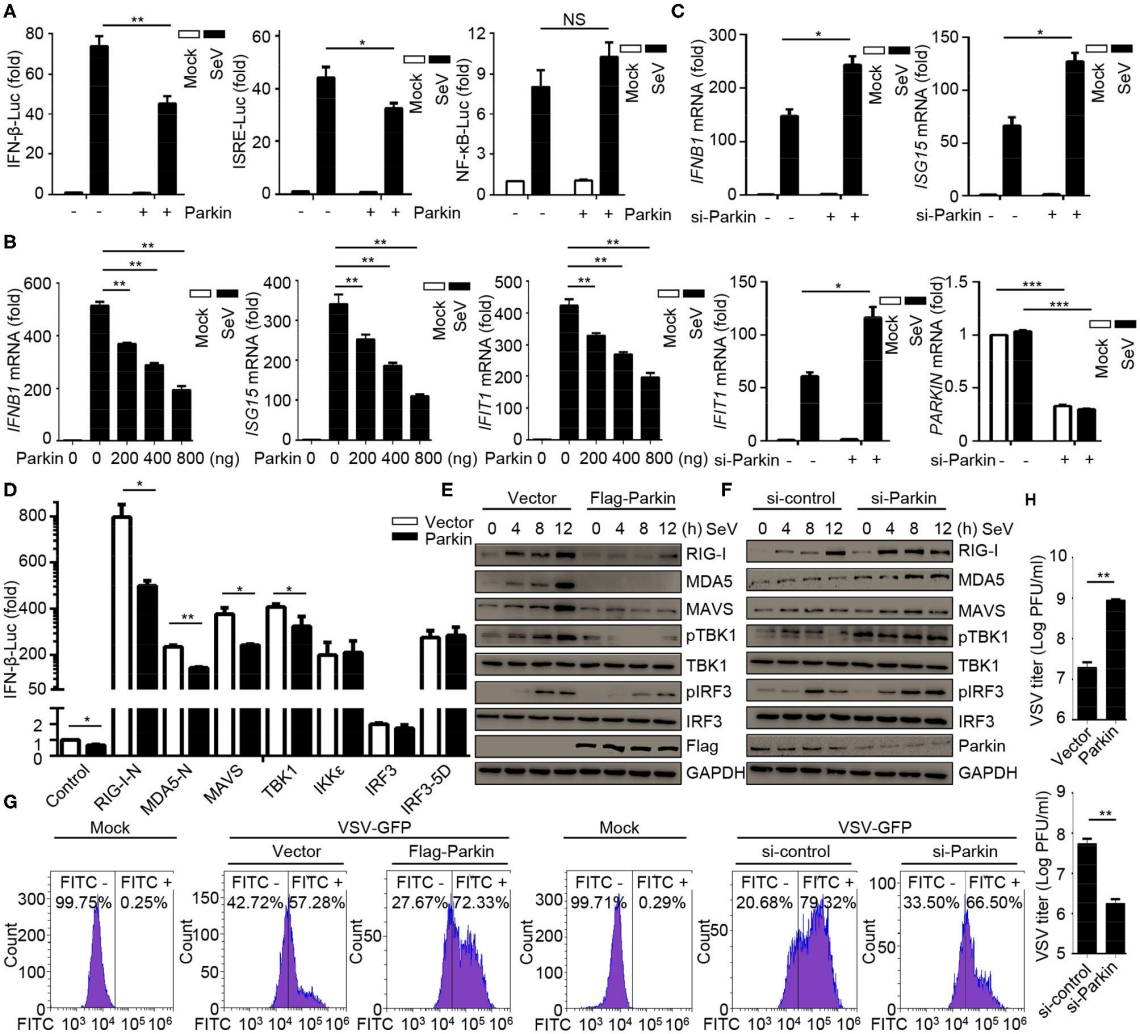
E3 ubiquitin-protein ligase Parkin inhibits type I IFN responses. **(A)** HEK293 cells were co-transfected with vector or Parkin and IFN-β, ISRE, or NF-κB luciferase reporter for 24 h, then unstimulated or stimulated with SeV for 10 h, and subjected to luciferase assays. **(B)** HEK293 cells were transfected with vector or different quality of Parkin for 24 h, then unstimulated or stimulated with SeV for 10 h, and subjected to quantitative RT-PCR analysis. **(C)** PMA-differentiated THP1 cells were transfected with si-control or si-Parkin for 48 h, then unstimulated or stimulated with SeV for 10 h, and subjected to quantitative RT-PCR analysis. **(D)** Vector or Parkin were co-transfected with IFN-β luciferase reporter and RIG-I-N, MDA5-N, MAVS, TBK1, IKKε, IRF3, or IRF3-5D for 24 h in HEK293 cells, then subjected to luciferase assays. **(E)** HEK293 cells were transfected with vector or Flag-Parkin for 24 h, then unstimulated or stimulated with SeV for different times, and the whole cell extracts were subjected to IB analysis. **(F)** HEK293 cells were transfected with si-control or si-Parkin for 48 h, then unstimulated or stimulated with SeV for different times, and the whole cell extracts were subjected to IB analysis. **(G)** HEK293 cells were transfected with vector or Parkin for 24 h, or transfected with si-control or si-Parkin for 48 h, then cells were infected with or without VSV-GFP (MOI = 0.01), and then cells were subjected to flow cytometer assays. **(H)** HEK293 cells were transfected with vector or Parkin for 24 h, or transfected with si-control or si-Parkin for 48 h, then cells were infected with VSV-GFP (MOI = 0.01), and the supernatants were subjected to VSV plaque assays. The data represent the average of three independent experiments and were analyzed by unpaired *t-*test. All data represent the mean ± S.D. *p < 0.05, **p < 0.01, and ***p < 0.001.

CCCP can promote the recruitment of Parkin to mitochondria, which eventually leads to mitophagy mediated by PINK1/Parkin. As PINK1 has been identified as a positive regulator for RLR signaling (31), we investigated the effects of Parkin in RLR-induced IFN-β signaling. Parkin was transfected into HEK293 cells, and the results suggested that Parkin could reduce SeV-induced activation of *IFNB1* promoter reporter and ISRE promoter reporter, instead of the NF-κB-responsive promoter reporter (Figure 2A). The expression of *IFNB1, ISG15*, and *IFIT1* mRNA were decreased in HEK293 cells transfected with Parkin upon infection with SeV (Figure 2B). Consistently, knockdown of Parkin could increase the expression of *IFNB1, ISG15*, and *IFIT1* mRNA in THP1, A549, and THP1 differentiated macrophages (Figure 2C and Supplementary Figures 2A,B). We then investigated which step of activation of IFN-β can be influenced by Parkin, and the results indicated that Parkin inhibited the activity of *IFNB1* promoter reporter induced by upstream activators, including RIG-I-N, MDA5-N, MAVS, and TBK1 (Figure 2D). Similarly, overexpression of Parkin decreased the expression of SeV-induced RIG-I, MDA5, MAVS, and the phosphorylation of TBK1 and IRF3 (Figure 2E). Additionally, knockdown of Parkin increased the expression of SeV-induced RIG-I, MDA5, MAVS, and the phosphorylation of TBK1 and IRF3 in HEK293 and A549 cells (Figure 2F and Supplementary Figure 2C). Moreover, overexpression of Parkin increased the level of VSV-GFP and VSV titers in HEK293 cells while knockdown of Parkin decreased the level of VSV-GFP and VSV titers (Figures 2G,H). In summary, our results conclude that E3 ubiquitin ligase Parkin negatively regulates RLR-mediated IFN-β signaling.

### Parkin Promotes the Inhibition of CCCP on Innate Immunity

**Figure 3:**
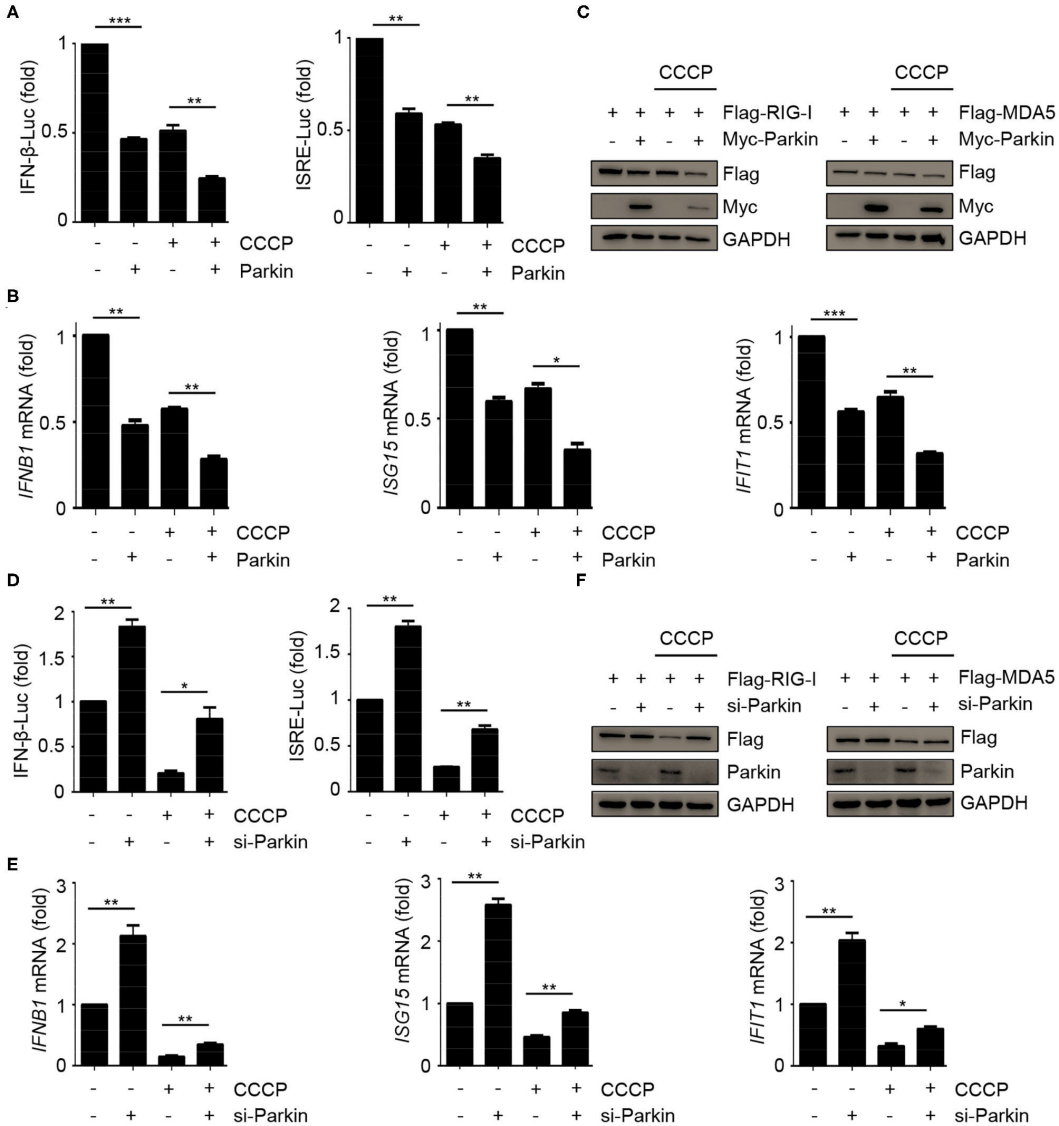
Parkin promotes the inhibition of CCCP on innate immunity. **(A)** HEK293 cells were co-transfected with vector or Parkin and IFN-β or ISRE luciferase reporter for 24 h, untreated or treated with CCCP (10 μM) for 2 h, then stimulated with SeV for 10 h, and subjected to luciferase assays. **(B)** HEK293 cells were transfected with vector or Parkin for 24 h, untreated or treated with CCCP (10 μM) for 2 h, then stimulated with SeV for 10 h, and subjected to quantitative RT-PCR analysis. **(C)** 293T cells were co-transfected with Flag-RIG-I or Flag-MDA5 and vector or Myc-Parkin for 24 h, untreated or treated with CCCP (10 μM) for 12 h, and the whole cell extracts were subjected to IB analysis. **(D)** HEK293 cells were co-transfected with si-control or si-Parkin and IFN-β or ISRE luciferase reporter for 48 h, untreated, or treated with CCCP (10 μM) for 2 h, then stimulated with SeV for 10 h, and subjected to luciferase assays. **(E)** HEK293 cells were si-control or si-Parkin for 48 h, untreated or treated with CCCP (10 μM) for 2 h, then stimulated with SeV for 10 h, and subjected to quantitative RT-PCR analysis. **(F)** 293T cells were co-transfected with Flag-RIG-I or Flag-MDA5 and si-control or si-Parkin for 48 h, untreated or treated with CCCP (10 μM) for 12 h, and the whole cell extracts were subjected to IB analysis. The data represent the average of three independent experiments and were analyzed by unpaired *t-*test. All data represent the mean ± S.D. *p < 0.05, **p < 0.01, and ***p < 0.001.

The above results have shown that CCCP and Parkin could inhibit the response of type I IFN. To confirm whether CCCP can inhibit the innate immunity-associated mitophagy mediated by PINK1/Parkin, we carried out a series of experiments. Luciferase assays revealed that Parkin could promote the inhibition of CCCP on SeV-induced activation of an *IFNB1* promoter reporter and ISRE promoter reporter (Figure 3A). Likewise, Parkin could increase the inhibitory effect of CCCP on the expression of *IFNB1, ISG15*, and *IFIT1* mRNA in HEK293 cells (Figure 3B). As a mitochondrial antiviral signaling protein, MAVS functions as a critical adaptor of the RLR signaling pathway. It has been reported that human parainfluenza virus type 3 (HPIV3)-mediated mitophagy inhibits the type I IFN response through MAVS (32). Parkin can also interact with MAVS and accumulate unanchored linear polyubiquitin chains on MAVS (33). In our study, we found that overexpression or knockdown of Parkin could change the expression of endogenous RIG-I and MDA5 but could not affect the protein level of endogenous MAVS (Figures 2E,F). Furthermore, CCCP did not affect the localization of MAVS on mitochondria (Supplementary Figure 3A). Therefore, our data indicate that Parkin affects innate immunity mainly through RIG-I and MDA5. CCCP and Parkin could both reduce the expression of exogenous RIG-I and MDA5. Notably, Parkin could increase the inhibitory effect of CCCP on the expression of exogenous RIG-I and MDA5 (Figure 3C). Nevertheless, siRNA-mediated knockdown of Parkin decreased the inhibition of CCCP on SeV-induced activation of an *IFNB1* promoter reporter and ISRE promoter reporter in HEK293 cells (Figure 3D). Similarly, knockdown of Parkin could decrease the inhibition of CCCP on the expression of *IFNB1, ISG15*, and *IFIT1* mRNA in HEK293 cells (Figure 3E). Also, knockdown of Parkin could decrease the inhibitory effect of CCCP on the expression of exogenous RIG-I and MDA5 (Figure 3F). These data suggest that the inhibitory effect of CCCP on innate immunity can be enhanced by Parkin.

### Mitophagy Mediated by PINK1/Parkin Negatively Regulates the Responses and Antiviral Immunity of Type I IFN

**Figure 4:**
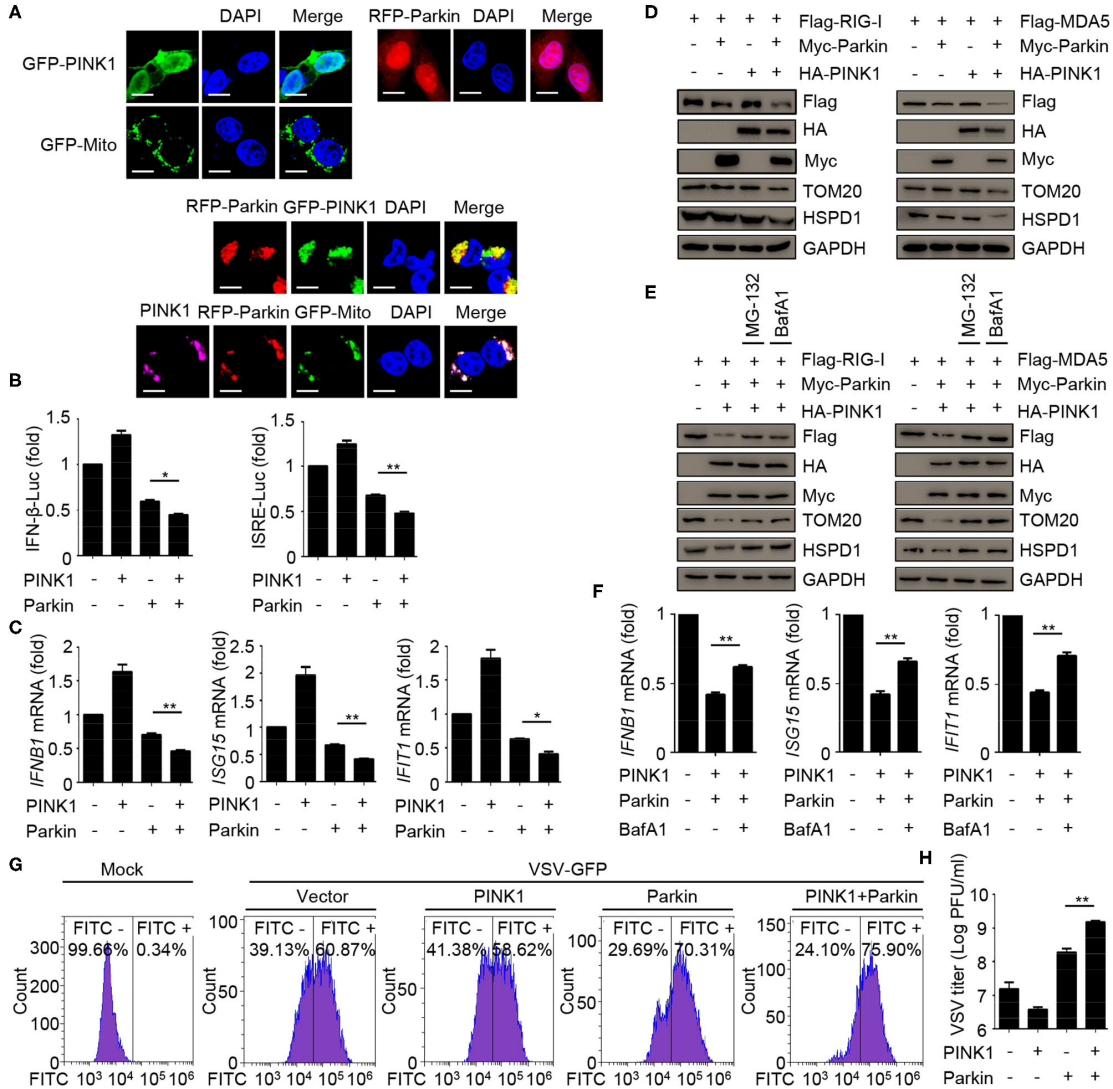
Mitophagy mediated by PINK1/Parkin negatively regulates the type I IFN responses. **(A)** 293T cells were transfected with PINK1, Parkin, and GFP-Mito alone or together for 24 h, and then subjected to IF assays. Scale bar is 10 μm. **(B)** HEK293 cells were transfected with PINK1 or Parkin alone or together with IFN-β or ISRE luciferase reporter, 24 h post-transfection, stimulated with SeV for 10 h, and subjected to luciferase assays. **(C)** HEK293 cells were transfected with PINK1 or Parkin alone or together, 24 h post-transfection, stimulated with SeV for 10 h, and subjected to quantitative RT-PCR analysis. **(D)** Flag-RIG-I or Flag-MDA5 were co-transfected with HA-PINK1 or Myc-Parkin in 293T cells for 24 h, and the whole cell extracts were subjected to IB analysis. **(E)** Flag-RIG-I or Flag-MDA5 were co-transfected with vector or HA-PINK1 and Myc-Parkin in 293T cells for 24 h, and untreated or treated with MG-132 (10 μM) or BafA1 (200 nM) for 8 h. The whole cell extracts were subjected to IB analysis. **(F)** HEK293 cells were transfected with vector or PINK1 and Parkin, 24 h post-transfection, stimulated with SeV for 10 h in the absence or presence of BafA1 (200 nM), and subjected to quantitative RT-PCR analysis. **(G)** HEK293 cells were transfected with PINK1 and Parkin alone or together for 24 h, then cells were infected with VSV-GFP (MOI = 0.01), and then cells were subjected to flow cytometer assays. **(H)** HEK293 cells were transfected with PINK1 and Parkin alone or together for 24 h, then cells were infected with VSV-GFP (MOI = 0.01), and the supernatants were subjected to VSV plaque assays. The data represent the average of three independent experiments and were analyzed by unpaired *t-*test. All data represent the mean ± S.D. *p < 0.05, and **p < 0.01.

To assess the relevance of mitophagy in innate immunity, we expressed PINK1 and Parkin alone or together, and evaluated the effect of mitophagy mediated by PINK1/Parkin on innate immunity. The results indicated that the single expression of PINK1 or Parkin expressed diffusely in the cells, and co-expression of PINK1 and Parkin could be recruited and co-localized with GFP-Mito (a mitochondrial marker) and accumulated in distinct puncta adjacent to damaged and incomplete mitochondria, an indicator of mitophagy (Figure 4A). Luciferase assays showed that PINK1 and Parkin could inhibit SeV-induced activation of an *IFNB1* promoter reporter and ISRE promoter reporter (Figure 4B). In addition, PINK1 and Parkin could reduce the expression of *IFNB1, ISG15*, and *IFIT1* mRNA in HEK293 cells (Figure 4C). We also observed that PINK1 and Parkin could reduce the expression of exogenous RIG-I and MDA5, and degrade the mitochondrial outer membrane protein TOM20 and the mitochondrial matrix protein HSPD1, an indication of mitophagy (Figure 4D). The proteasome inhibitor MG-132 and autophagy inhibitor bafilomycin A1 (BafA1) could reduce the inhibition of PINK1 and Parkin on RIG-I and MDA5 (Figure 4E). Furthermore, autophagy inhibitor BafA1 could reduce the inhibition of PINK1 and Parkin on the expression of *IFNB1, ISG15*, and *IFIT1* mRNA in HEK293 cells (Figure 4F). Moreover, overexpression of PINK1 and Parkin increased the level of VSV-GFP and VSV titers in HEK293 cells (Figures 4G,H). Collectively, these data suggest that mitophagy mediated by PINK1/Parkin can suppress the antiviral immune responses.

### Parkin Interacts With RIG-I and MDA5

**Figure 5:**
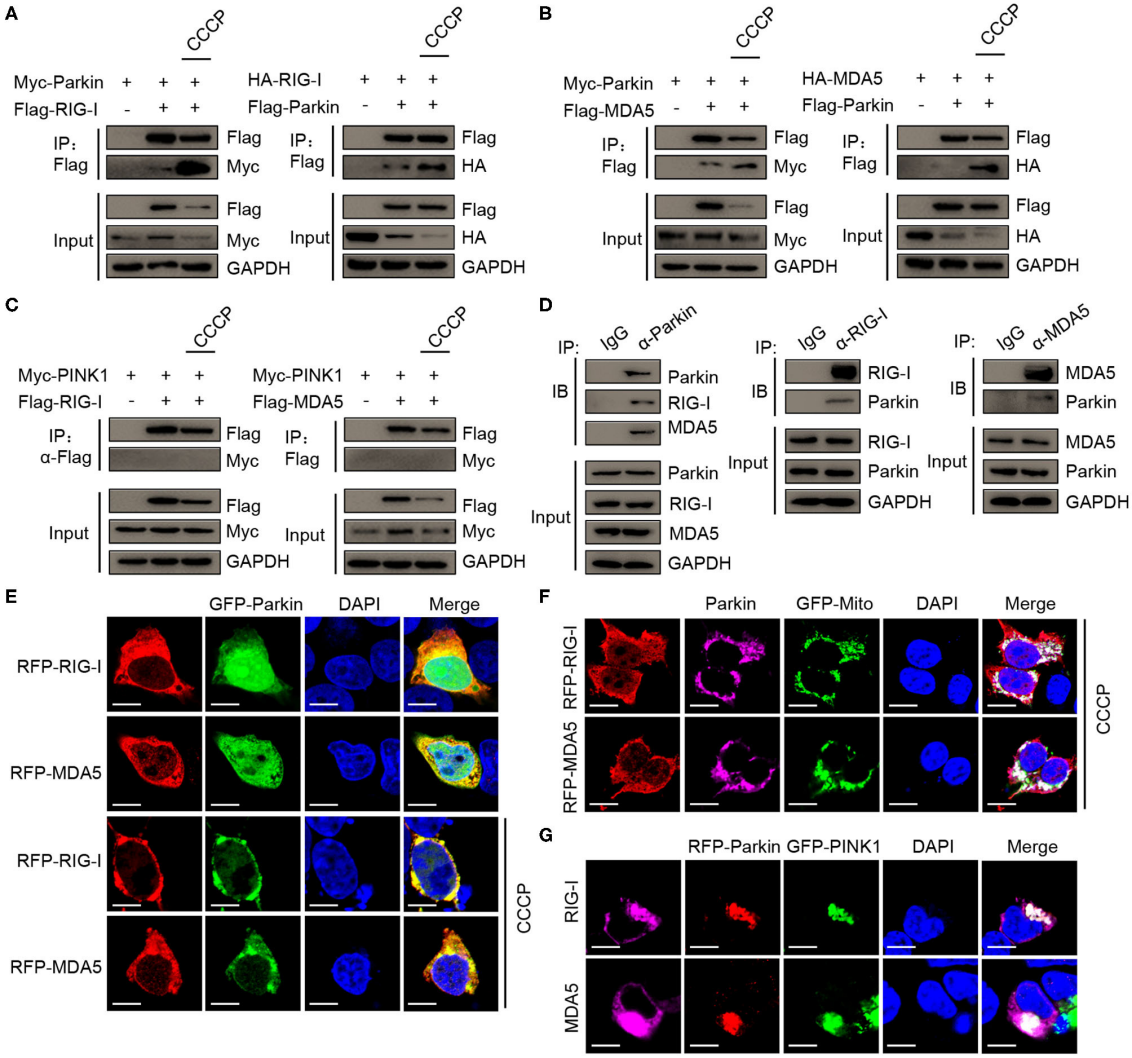
CCCP promotes the interaction between Parkin and RIG-I or MDA5 on mitochondria. **(A)** 293T cells were co-transfected with Myc-Parkin and vector or Flag-RIG-I, or co-transfected with HA-RIG-I and vector or Flag-Parkin. 24 h post-transfection, untreated or treated with CCCP (10 μM) for 12 h, and then subjected to IP and IB analysis. **(B)** 293T cells were co-transfected with Myc-Parkin and vector or Flag-MDA5, or co-transfected with HA-MDA5 and vector or Flag-Parkin. 24 h post-transfection, untreated, or treated with CCCP (10 μM) for 12 h, and then subjected to IP and IB analysis. **(C)** 293T cells were co-transfected with Myc-PINK1 and vector, Flag-RIG-I, or Flag-MDA5. 24 h post-transfection, untreated or treated with CCCP (10 μM) for 12 h, and then subjected to IP and IB analysis. **(D)** HEK293 cells were stimulated with SeV for 10 h, and then whole cell lysates were collected, immunoprecipitated with indicated antibodies and subjected to IB analysis. **(E)** 293T cells were co-transfected with GFP-Parkin and RFP-RIG-I, or RFP-MDA5. 24 h post-transfection, untreated, or treated with CCCP (10 μM) for 6 h, and then subjected to IF analysis. Scale bar is 10 μm. **(F)** Parkin and GFP-Mito were co-transfected with RFP-RIG-I or RFP-MDA5 for 24 h in 293T cell, treated with CCCP (10 μM) for 6 h, and then subjected to IF analysis. Scale bar is 10 μm. **(G)** GFP-PINK1 and RFP-Parkin were co-transfected with RIG-I or MDA5 for 24 h in 293T cells, then subjected to IF analysis. Scale bar is 10 μm.

Next, we sought to determine the molecular mechanisms of mitophagy mediated by PINK1/Parkin decreasing type I interferon signaling. The co-immunoprecipitation assays showed that Parkin could interacted with RIG-I, and CCCP could promote the interaction (Figure 5A). We also found the interaction between Parkin and MDA5, and CCCP could also promote the interaction (Figure 5B). In addition, we did not observe the interaction between PINK1 and RIG-I or MDA5 in 293T cells treated with CCCP (Figure 5C). Furthermore, we found that endogenous interaction between Parkin and RIG-I or MDA5 in HEK293 cells stimulated with SeV (Figure 5D). Under a microscope, we observed the colocalization of Parkin and RIG-I or MDA5, and CCCP could promote their colocalization on mitochondria (Figures 5E,F). Moreover, PINK1 could promote the colocalization of Parkin and RIG-I or MDA5 (Figure 5G). These results indicate that Parkin interacts with RIG-I and MDA5, and the interaction can be enhanced by CCCP.

### Parkin Increases K48-Linked Polyubiquitination of RIG-I and MDA5

**Figure 6:**
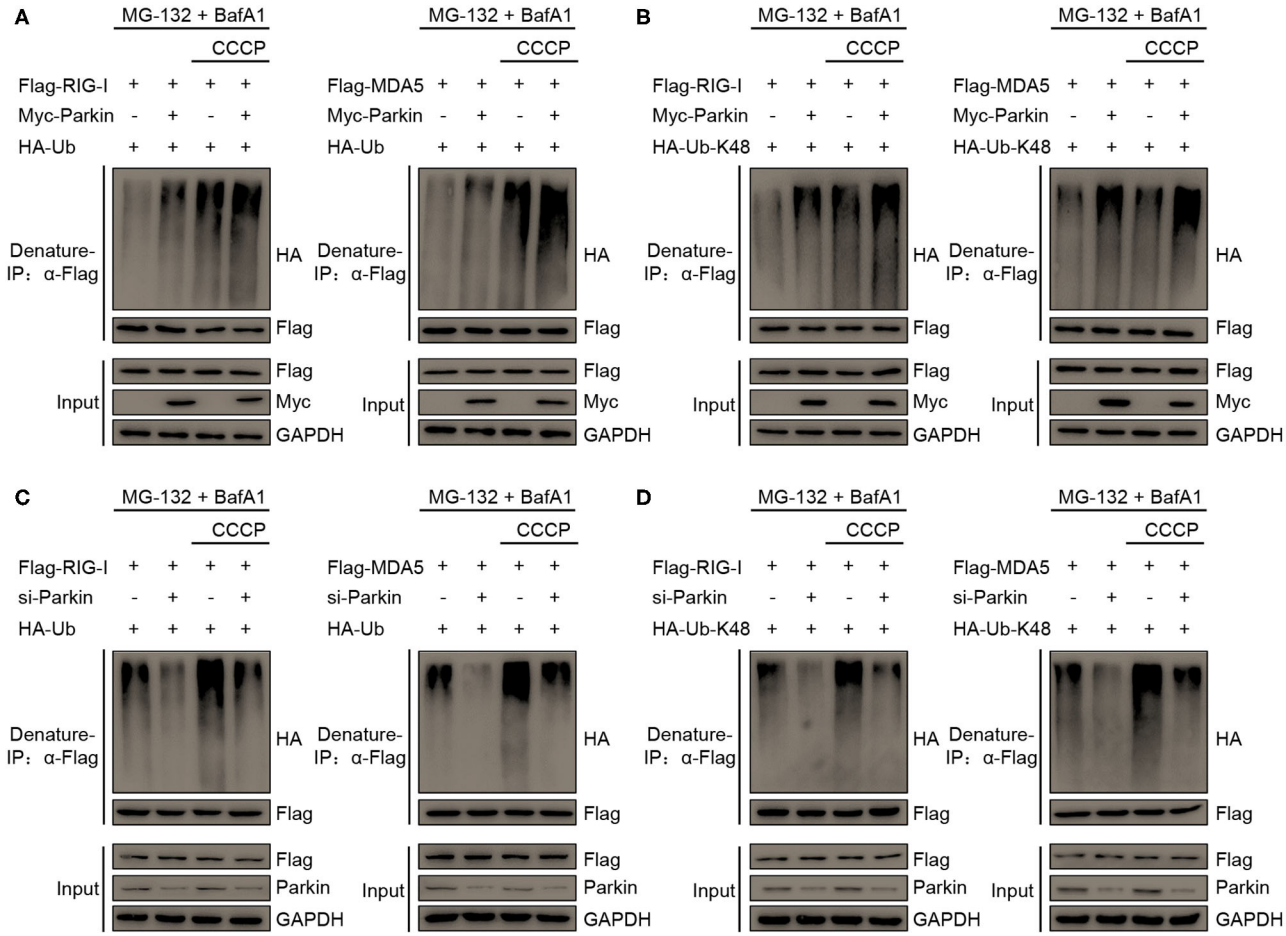
Parkin catalyzes the K48-linked polyubiquitination of RIG-I and MDA5. **(A)** HA-Ubiquitin and Flag-RIG-I or Flag-MDA5 were co-transfected with vector or Myc-Parkin in 293T cells. 24 h post-transfection, untreated or treated with CCCP (10 μM) for 12 h in the presence of MG-132 (10 μM) and BafA1 (200 nM) at the last 8 h, and then subjected to denature-IP and IB analysis. **(B)** HA-Ubiquitin-K48 and Flag-RIG-I or Flag-MDA5 were co-transfected with vector or Myc-Parkin in 293T cells. 24 h post-transfection, untreated or treated with CCCP (10 μM) for 12 h in the presence of MG-132 (10 μM) and BafA1 (200 nM) at the last 8 h, and then subjected to denature-IP and IB analysis. **(C)** HA-Ubiquitin and Flag-RIG-I or Flag-MDA5 were co-transfected with si-control or si-Parkin in 293T cells. 48 h post-transfection, untreated or treated with CCCP (10 μM) for 12 h in the presence of MG-132 (10 μM) and BafA1 (200 nM) at the last 8 h, and then subjected to denature-IP and IB analysis. **(D)** HA-Ubiquitin-K48 and Flag-RIG-I or Flag-MDA5 were co-transfected with si-control or si-Parkin in 293T cells. 48 h post-transfection, untreated or treated with CCCP (10 μM) for 12 h in the presence of MG-132 (10 μM) and BafA1 (200 nM) at the last 8 h, and then subjected to denature-IP and IB analysis.

To investigate whether Parkin regulates the polyubiquitination of RIG-I and MDA5 through its E3 ligase activity, we designed a series of experiments. We found that Parkin and CCCP could both increase the polyubiquitination of RIG-I or MDA5, and Parkin could increase the CCCP-mediated polyubiquitination of RIG-I or MDA5 in the presence of MG-132 and BafA1 (Figure 6A). K63-linked polyubiquitination is required for the activation of RIG-I and MDA5 upon viral infection and K48-linked polyubiquitination is indicated for the degradation of RIG-I and MDA5. To further investigate the type of Parkin-mediated polyubiquitination of RIG-I or MDA5, we used vectors expressing HA-Ubiquitin-K48, which contain arginine to replace all lysine residues except lysine at position 48. It was revealed by immunoprecipitation experiments that Parkin and CCCP could both increase the K48-linked polyubiquitination of RIG-I or MDA5, and Parkin could increase CCCP-mediated K48-Ubiquitination of RIG-I or MDA5 in the presence of MG-132 and BafA1 (Figure 6B). In addition, siRNA-mediated knockdown of Parkin substantially attenuated RIG-I or MDA5 polyubiquitination in 293T cells transfected with HA-Ubiquitin or HA-Ubiquitin-K48 in the presence of MG-132 and BafA1 (Figures 6C,D). In summary, our results depict the molecular mechanism that Parkin and CCCP promote K48-linked polyubiquitination of RIG-I and MDA5, and Parkin could increase the effect of CCCP.

## Discussion

Evidence accumulated during the last few years has convincingly revealed that the immune system is excessively activated in the brains of patients with Parkinson's disease (34). Moreover, the latest researches show that Parkinson's disease may be related to the overactivation of patients' autoimmune response (35). There are a set of pro-inflammatory cytokines including IL-6, TNF, IL-1β, and IFN-γ in the serum of patients with Parkinson's disease (36). What's more, familial Parkinson's disease can be induced by the mutations of PINK1 and Parkin, which leads to mitophagy dysfunction. The dysfunction of mitophagy may excessively activate the immune system of Parkinson's disease (37). Here, we provided several lines of evidence to demonstrate that mitophagy induced by CCCP negatively regulates the innate antiviral immunity.

As a special autophagy pathway, mitophagy mediates the clearance of damaged mitochondria by lysosomes, which plays an important role in mitochondrial quality control (38). In addition, mitochondrial stress can lead to the release of damage-associated molecular patterns (DAMPs), which can activate innate immunity. Furthermore, mitochondrial DNA (mtDNA) can be recognized by the cGAS/STING-dependent DNA sensing pathway, which initiates the innate immunity (39). Mitochondrial double-stranded RNA engages an MDA5-driven antiviral signaling pathway that triggers the response of type I interferon (40). Mitochondrial apoptosis is mediated by BAK and BAX, which triggers the release of mitochondrial DNA, and apoptotic caspases suppresses the production of type I interferon (41–43). Mitophagy is a form of selective autophagy, PINK1 and Parkin are the two key molecules in the regulation of mitophagy (28, 44). These studies suggest that PINK1 and Parkin mediated mitophagy can mitigate inflammation and autoimmune disease (36, 45, 46). When neurodegenerative diseases occur in the central nervous system, mitochondrial DNA can activate the innate immunity and cause neuroinflammation (47). Type I IFN contribute to the inflammatory response during normal aging and in age-related neurodegenerative disorders (48–51). In our study, we found that PINK1 and Parkin mediated mitophagy can inhibit the response and antiviral immunity of type I IFN, and these results may suggest that loss of functional PINK1 and Parkin may increase IFN production and drive the pathology of neurodegeneration.

Recognition of the RNA by endosomal and cytosolic sensors is a central element in the detection of virus by the innate immune system. The RNA sensors RIG-I and MDA5 recognize the dsRNA and activate the innate antiviral immunity subsequently (52). According to recent reports, polyubiquitination regulates the activation of RIG-I and MDA5 so as to avoid their sustained activation. It has been identified that several E3 ligases promote K48-linked ubiquitination of RIG-I and MDA5, including RNF125, Siglec-G, TRIM40, and STUB1 (53–56). Here, we demonstrate that Parkin served as a specific regulator of RIG-I-mediated and MDA5-mediated innate antiviral immunity through direct conjugation of K48-linked polyubiquitin chains to RIG-I and MDA5. Therefore, Parkin may inhibit the innate immunity by enhancing the degradation of RIG-I and MDA5.

In summary, we have identified an E3 ubiquitin ligase, Parkin, as a negative regulator of RIG-I and MDA5. Parkin interacted with RIG-I and MDA5, and then catalyzed the K48-linked polyubiquitination of RIG-I and MDA5, which facilitated the degradation of RIG-I and MDA5 and prevented excessive activation of innate antiviral immunity.

## Materials and Methods

### Cell Culture and Viruses

293T, HEK293, Raw264.7, A549, and VERO cells were maintained in DMEM medium (Hyclone) supplemented with 10% heat-inactivated fetal bovine serum (FBS, Gibco) and cultured at 37°C in a 5% CO_2_ incubator. BMDCs were isolated from mouse tibia and femur as described previously (57). THP-1 cells were maintained in 1640 medium (Hyclone) supplemented with 10% heat-inactivated fetal bovine serum (FBS, Gibco) and cultured at 37°C in a 5% CO_2_ incubator. THP-1 cells were differentiated to macrophages for 48 h with 50 ng/ml phorbol myristate acetate (PMA). Sendai virus (SeV) and vesicular stomatitis virus (VSV) carrying a GFP reporter gene (VSV-GFP) were kindly provided by Dr Hong-Bing Shu.

### Reagents and Antibodies

The chemical reagents used in this study were listed as follows: CCCP (C2759), MG-132 (M8699), PMA (P1585), and bafilomycin A1 (B1793) were purchased from Sigma. Anti-Parkin antibody (4211), anti-RIG-I (3743) antibody, anti-MDA5 (5321) antibody, anti-MAVS (24930) antibody, anti-pTBK1-Ser172 (5483) antibody, anti-TBK1 (38066) antibody, anti-pIRF3-Ser396 (29047) antibody, anti-IRF3 (11904) antibody, anti-TOM20 (42406) antibody, anti-HSPD1 (12165) antibody, and anti-GAPDH (5174) were purchased from Cell Signaling Technology. Mouse anti-HA antibody (H3663), rabbit anti-HA antibody (H6908), mouse anti-Myc antibody (SAB2702192), rabbit anti-Myc antibody (C3956), mouse anti-Flag antibody (F3165), and rabbit anti-Flag antibody (F7425) were purchased from Sigma. Anti-Flag M2 Affinity Gel (A2220) were purchased from Millipore. Goat anti-rabbit Alexa Fluor Plus 647 (A32733) were purchased from Invitrogen.

### Plasmids

pEF-Flag-RIG-I, pEF-Flag-MDA5, pEF-Flag-MAVS, pEF-Flag-MDA5-N, pEF-Flag-RIG-I-N, pEF-Flag-TBK1, pEF-Flag-IKKε, pEF-Flag-IRF3, pEF-Flag-IRF3-5D, pRK-HA-RIG-I, and pRK-HA-MDA5 have described previously (58). pRK5-HA-Parkin (#17613) and pCMVTNT-PINK1-N-myc (#13313) were purchased from Addgene. pEF-Flag-Parkin, pEF-Myc-Parkin, pEF-Myc-PINK1, pRK-HA-PINK1, pEGFP-Parkin, pDs-Red-Parkin, pEGFP-PINK1, pDs-Red-MAVS, pDs-Red-RIG-I, and pDs-Red-MDA5 are constructed according to the manufacturer's standard procedures. GFP-Mito plasmid was purchased from Invitrogen (catalog number: V822-20). HA-Ub, HA-Ub-K48 have described previously (57).

### RNA Interference Assay

Human Parkin-specific siRNA and control siRNA were obtained from Ribobio, the siRNA sequence used in this study are as following: si-Parkin 5′-GGAGUGCAGUGCCGUAUUU-3′. These siRNA duplexes were transfected into cells using RNAi MAX (Invitrogen) according to the manufacturer's protocol.

### Quantitative Real-Time PCR

Total RNA was isolated from HEK293 or Raw264.7 cells using TRIzol reagent (Invitrogen) and then was reverse transcribed into cDNA using PrimeScript RT reagent kit with gDNA Eraser (Takara) according to the manufacturer's instructions. An ABI 7300 Detection System (Applied Biosystems) and a SYBR RT-PCR kit (Takara) were used to amplify the reverse-transcription products of different samples for quantitative RT-PCR analysis. The RT-PCR primer of Parkin is given as following: Parkin forward, 5′-GTGTTTGTCAGGTTCAACTCCA-3′ and Parkin reverse, 5′-GAAAATCACACGCAACTGGTC-3′. Other primer sequences were as reported in our previous work (58).

### Luciferase Assay

HEK293 cells (2 × 10^5^) were seeded on 24-well-plates and transfected with a mixture of renilla luciferase plasmid and IFN-β, ISRE, or NF-κB luciferase reporter plasmid together with indicated expression plasmids or empty control plasmid. After 24–36 h of transfection, luciferase activity was measured with a dual-luciferase reporter assay system according to the manufacturer's instructions (Promega). By calculating the ratio of firefly luciferase activity to renilla luciferase activity, data were normalized for transfection efficiency.

### Flow Cytometry

HEK293 cells were seeded and transfected with plasmids or siRNA for 48 h, then infected with VSV-GFP for 2 h. Then cells were washed three times with PBS and added fresh medium for 24 h, cells were immediately analyzed by the flow cytometer (BD FACSAria III, BD).

### VSV Plaque Assay

HEK293 cells were seeded and transfected with plasmids or siRNA for 48 h, then infected with VSV-GFP for 2 h. Then cells were washed three times with PBS and added fresh medium for 24 h, collected supernatants, and diluted to infect VERO cells seeded on 24-well-plates. Two hours later, supernatants were removed, and methylcellulose was added. Three days later, cells were stained with crystal violet (0.2%) overnight. The results were averaged and multiplied by the dilution factor to calculate the viral titer as PFU/ml.

### Immunofluorescence (IF) Assay

293T cells (3 × 10^4^) were seeded in chamber slides and transfected with plasmids for 24 h. Then the cells were fixed with 4% formaldehyde for 10 min and permeabilized with 0.1% Triton X-100 for 10 min. Then cells were blocked with 5% BSA for 30 min. The samples were incubated with primary antibodies overnight at 4°C. Next, cells were incubated with appropriate second antibodies for 1 h at 37°C, then conducted with 4,6-diamidino-2-phenylindole (DAPI) for 5 min. The confocal images were captured using Zeiss LSM780 microscope.

### Immunoprecipitation (IP) and Immunoblotting (IB) Analysis

For immunoprecipitation experiments, 293T cells (5 × 10^6^) were seeded on 10-cm dishes, then transfected with a total of 12 μg appropriate plasmids. After 24 h, cells were lysed with lysis buffer (50 mM Tris-HCl (pH 7.6), 150 mM NaCl, 10 mM NaF, 2 mM EGTA, 2 mM DTT, 1 mM Na_3_VO_4_, and 0.5% Triton-X-100 and 1 × Cocktail). Then immunoprecipitated with 30 μL anti-Flag agarose (Millipore) at 4°C overnight. Beads were washed four times with RIPA buffer, and immunoprecipitates were eluted with 2 × SDS loading buffer by boiling for 5 min and subjected to IB analysis. For immunoblotting experiments, cells were washed and lysed in RIPA buffer, cell extracts were centrifugated at 12,000 rpm for 10 min, then mixed with 5 × SDS loading buffer and boiled for 5 min. Samples were resolved on 10% SDS-PAGE gels and subjected to western blotting that was performed according to the instructions as indicated.

### *In vivo* Ubiquitination Assay

Whole cells were lysed with lysis buffer (100 μl) and the supernatants were denatured at 100°C for 5 min in the presence of 1% SDS by lysates. The denatured lysates were diluted with lysis buffer until the concentration of SDS was reduced < 0.1% followed by immunoprecipitation (denature-IP) with the indicated antibodies. The immunoprecipitants were subject to immunoblot analysis.

### Statistical Analysis

The data were shown as the mean ± standard deviation (mean ± SD) from at least three independent experiments. The statistical significance of the difference between any two samples was evaluated by Student's *t*-test using GraphPad Prism for Windows version 5.0 (GraphPad Software, USA). The values of *p* < 0.05 were considered statistically significant.

## Data Availability Statement

All datasets generated for this study are included in the article/Supplementary Material.

## Author Contributions

DG, C-ML, and LB conceived and designed the experiments. LB carried out the experiments and analyzed data. HW, PH, SG, MH, JX, PL, YZ, PJ, YC, GL, and CY assisted with the experiments. LC assisted with experimental design. DG, LB, and HW wrote the manuscript. All authors commented on the manuscript.

## Conflict of Interest

The authors declare that the research was conducted in the absence of any commercial or financial relationships that could be construed as a potential conflict of interest.
