# Supplementary material for: The Ubiquitin E3 Ligase Parkin Inhibits Innate Antiviral Immunity Through K48-Linked Polyubiquitination of RIG-I and MDA5
Source: Front Immunol. 2020 Sep 2;11:1926. doi: 10.3389/fimmu.2020.01926 (PMC7492610; doi:10.3389/fimmu.2020.01926)
Supplement: Supplementary file 1 [file Image_1.pdf]

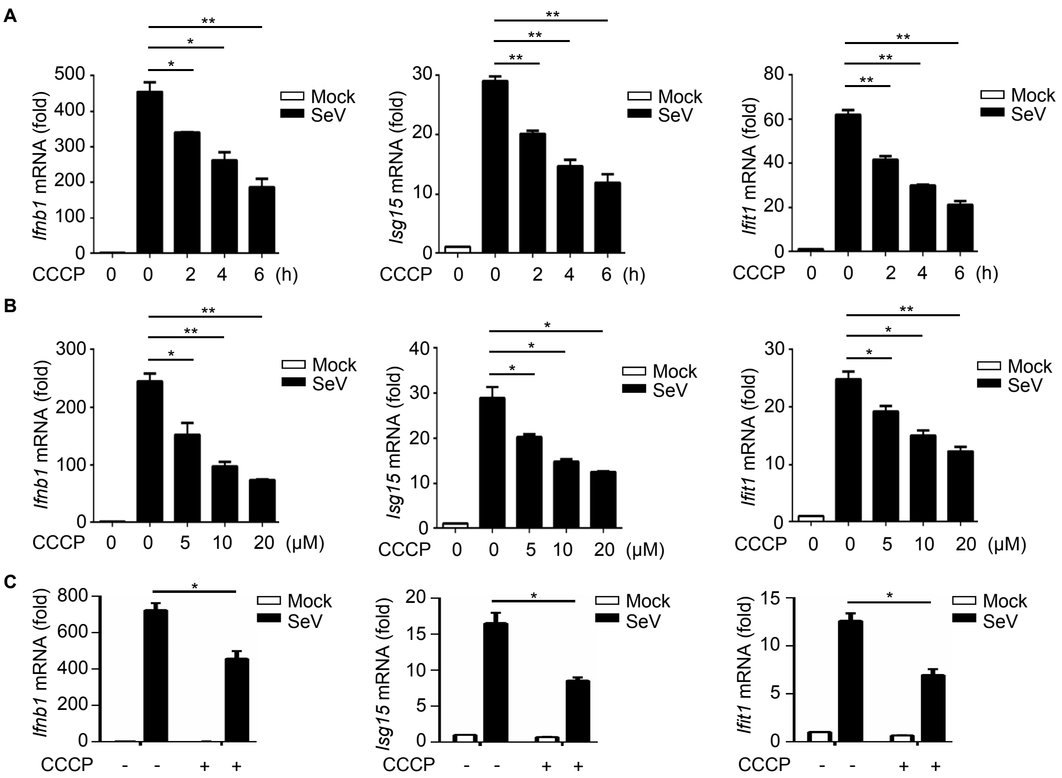

SUPPLEMENTARY FIGURE 1. Mitochondrial uncoupler CCCP inhibits type I IFN responses.

(A) Raw264.7 cells were untreated or treated with CCCP (10 μM) for different times, unstimulated or stimulated with SeV for 10 h. Then quantitative RT-PCR analysis of *Ifnb1*, *Isg15*, and *Ifit1* mRNA. **(B)** Raw264.7 cells were untreated or treated with different concentrations of CCCP for 2 h, then unstimulated or stimulated with SeV for 10 h, and subjected to quantitative RT-PCR analysis. **(C)** BMDCs were untreated or treated with CCCP (10 μM) for 2 h, then unstimulated or stimulated with SeV for 10 h, and subjected to quantitative RT-PCR analysis. The data represent the average of three independent experiments and were analyzed by unpaired *t* test. All data represent the mean ± S.D. *$p < 0.05$, **$p < 0.01$, ***$p < 0.001$.

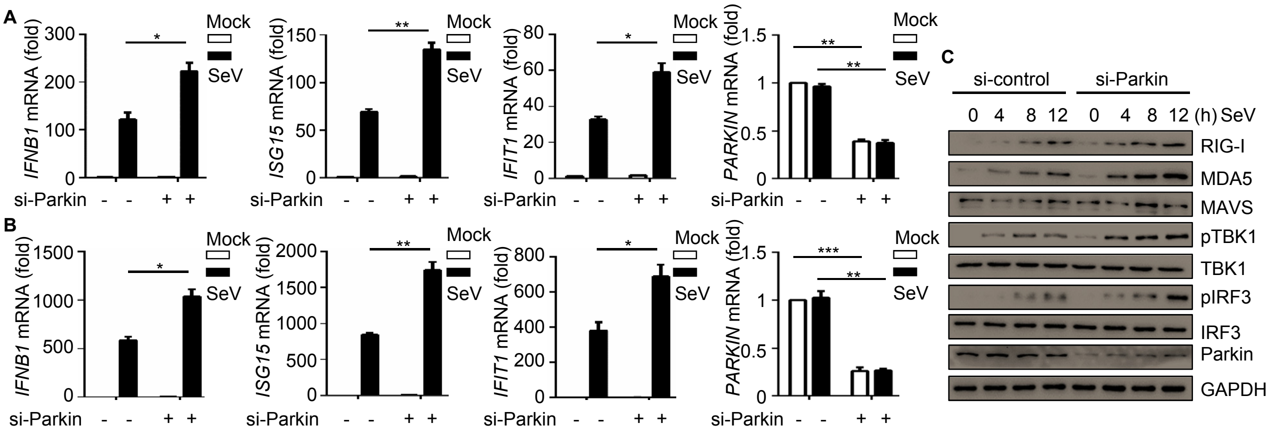

SUPPLEMENTARY FIGURE 2. Knockdown of Parkin increases type I IFN responses.

(A) THP1 cells were transfected with si-control or si-Parkin for 48 h, then unstimulated or stimulated with SeV for 10 h, and subjected to quantitative RT-PCR analysis. **(B)** A549 cells were transfected with si-control or si-Parkin for 48 h, then unstimulated or stimulated with SeV for 10 h, and subjected to quantitative RT-PCR analysis. **(C)** A549 cells were transfected with si-control or si-Parkin for 48 h, then unstimulated or stimulated with SeV for different times, and the whole cell extracts were subjected to IB analysis. The data represent the average of three independent experiments and were analyzed by unpaired *t* test. All data represent the mean ± S.D. *$p < 0.05$, **$p < 0.01$, ***$p < 0.001$.

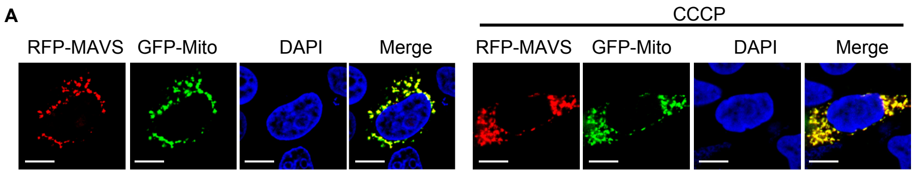

SUPPLEMENTARY FIGURE 3. CCCP does not affect the localization of MAVS.

(A) 293T cells were co-transfected with RFP-MAVS and GFP-Mito. 24 h post-transfection, untreated or treated with CCCP (10 μM) for 6 h, and then subjected to IF analysis. Scale bar is 10 μm.
